# Validity and reliability of the Manchester Oxford Foot Questionnaire (MOXFQ) in one-year postoperative ankle fracture patients—a validation study

**DOI:** 10.1186/s41687-025-00845-w

**Published:** 2025-02-05

**Authors:** Michael Quan Nguyen, Marjolein Memelink Iversen, Knut Harboe, Ingvild Dalen, Aksel Paulsen

**Affiliations:** 1 Department of Quality and Health Technology, Faculty of Health Sciences, University of Stavanger, Stavanger, Norway; 2 Department of Orthopedic Surgery, Stavanger University Hospital, Helse Stavanger HF, Stavanger, Norway; 3 The Fracture Registry of Western Norway, Helse Vest RHF, Department of Orthopedic Surgery, Stavanger University Hospital, Stavanger, Norway; 4 Department of Health and Caring Sciences, Faculty of Health and Social Sciences, Western Norway University of Applied Sciences, Bergen, Norway; 5 Centre on Patient-reported Outcomes, Department of Research and Development, Haukeland University Hospital, Helse Bergen HF, Bergen, Norway; 6 Department of Clinical Medicine, Faculty of Medicine, University of Bergen, Bergen, Norway; 7 Department of Research, Stavanger University Hospital, Helse Stavanger HF, Stavanger, Norway; 8 Department of Public Health, Faculty of Health Sciences, University of Stavanger, Stavanger, Norway

**Keywords:** Ankle fractures, Patient reported outcome measures, Validation study, Structural validity, Reliability

## Abstract

**Background:**

Ankle fracture patients are a heterogenous group with differences in age, sex, fracture morphology, and treatment provided. With the increased focus on patient-centered treatment, patient-reported outcome measures (PROMs) are increasingly adopted by clinicians to facilitate best clinical practice. The Manchester Oxford Foot Questionnaire (MOXFQ) has demonstrated good measurement properties when used in patients with foot or ankle disease. The PROM has three domains: (1) Pain; (2) Walking/Standing; and (3) Social Interaction. One study found sufficient content validity for the Pain and Walking/Standing domains when used in the evaluation of ankle fracture patients. Another validation study demonstrated acceptable structural validity and reliability for the MOXFQ in ankle fracture patients 12 weeks after injury. The aim of this study is to assess the structural validity and reliability of the Norwegian version of the MOXFQ in the context of an ankle fracture patients one year after surgery and provide patient acceptable symptom state (PASS) estimates.

**Methods:**

A pragmatic cross-sectional study design was used to collect the one-year MOXFQ follow-up data from patients surgically treated for an ankle fracture in the period 2017 to 2020 at (Stavanger University Hospital). The structural validity and internal consistency were assessed using confirmatory factor analysis. A separate test-retest study including patients at least one year since ankle surgery was used in the assessment of reliability and measurement error.

**Results:**

A confirmatory factor analysis of the three-factor model of the MOXFQ had a good model fit (TLI 0.94; CFI 0.95; RMSEA 0.094; SRMR 0.039). However, the measurement model demonstrated poor discriminant validity of the three factors. A unidimensional model of the 16 items had worse model fit, while a second-order factor model demonstrated strong factor loadings for a second-order factor. A bi-factor model also revealed a strong general factor but also unique variance in the Pain and Social Interaction domain. The domains had good internal consistency (McDonald’s omega 0.80 to 0.95) and test-retest reliability (ICC 0.80 to 0.92). The standard errors of measurements for the three domains were between 6.5 and 7.5, and 5.5 for the MOXFQ-Index (scale 0 to 100). PASS estimates for the (sub)scales were: Pain 45; Walking/Standing 39; Social Interaction 19; and MOXFQ-Index 34.

**Conclusion:**

The MOXFQ with three domains demonstrated sufficient structural validity and reliability when used in the evaluation of a one-year postoperative ankle fracture population. Reporting the scores of the Pain and Walking/Standing domains was best supported.

**Supplementary Information:**

The online version contains supplementary material available at 10.1186/s41687-025-00845-w.

## Background

About 10% of patients with fractures admitted to the emergency ward are ankle fracture patients [1, 2]. This heterogenous patient group may present with a unimalleolar, bimalleolar or trimalleolar fracture, where multi-malleolar fractures are more common in elderly patients [3, 4]. The management of ankle fractures is usually tailored to the specific patient, including the assessment of the fracture morphology, patients’ biology, and choice of implants available [5]. The Arbeitsgemeinschaft für Osteosynthesefragen / Orthopaedic Trauma Association (AO/OTA) fracture classification [6] is often used for the classification of fractures by assigning numbers and letters to describe the location and morphology of the fracture. Ankle fractures are classified as 44. In the follow-up of ankle fractures, the assessment of pain, range of motion and radiologic examinations are routinely performed. However, there has been an increased focus on patient-centered care the past decades and the use of patient-reported outcome measures (PROMs) have attained widespread recognition [7].

A patient reported outcome (PRO) *is any report of the status of a patient’s health condition that comes directly from the patient*,* without interpretation of the patient’s response by a clinician or anyone else. The outcome can be measured in absolute terms (e.g.*,* severity of a symptom*,* sign*,* or state of a disease) or as a change from a previous measure. In clinical trials*,* a PRO instrument can be used to measure the effect of a medical intervention on one or more concepts (i.e.*,* the thing being measured*,* such as a symptom or group of symptoms*,* effects on a particular function or group of functions*,* or a group of symptoms or functions shown to measure the severity of a health condition)* [8]. Selecting a PROM that is validated and accommodates good measurement properties for the purpose of use and for the specific condition is important to assure that the PROM measures what it is intended to measure. The COnsensus-based Standards for the selection of health Measurement INstruments (COSMIN)-group defines three domains in the assessment of the measurement properties of an instrument: (1) validity, i.e. *the degree to which the instrument measures the construct it purports to measure*, (2) reliability, i.e. *the degree to which the measurement is without measurement error*, and (3) responsiveness, i.e. *the ability of a PROM to detect change over time in the construct to be measured* [9]. New validation studies are necessary to ensure the performance of the instrument when changing the context of use. The process of determining sufficient validity of an instrument includes the assessment of *content validity*, which is performed using qualitative methods [10], and *construct validity*, which includes the evaluation of the structural validity of the instrument [9], i.e. if *the scores of a PROM are an adequate reflection of the dimensionality of the construct to be measured*. In instances where validity studies indicate insufficient content validity, e.g. when a multidimensional instrument fails to comprehensively measure the intended construct, assessing structural validity would not add to establish construct validity. However, multidimensional PROMs may include subscales that measure relevant constructs when applied to a different context, e.g., in settings when there is a lack of validated PROMs for a specific conditions and clinicians are restricted to using PROMs that has only been validated for similar conditions. Exploring the structure would then provide information on whether reporting scores of relevant domains are justifiable or not [11]. A factor analysis would also assess the degree of unidimensionality of the domains, i.e. if the items in a (sub)scale measure the same construct, which is a requirement for assessing reliability [12]. Moreover, the determination of reliability includes calculation of measurement error parameters to provide the users with clinically useful estimates. Complementing with clinical cut-off values, e.g., patient acceptable symptom state (PASS), enhances the utilization of PROMs as a clinical tool [13].

A recent systematic review of measurement properties of PROMs identified 50 PROMs used in patients with foot or ankle disease where the Manchester Oxford Foot Questionnaire (MOXFQ) had the best measurement properties [14]. The MOXFQ was initially developed to be used as an evaluative instrument in patients with hallux valgus foot deformity [15]. The developers of the MOXFQ extended the use of the instrument to include patients with ankle problems by altering the wording in the questionnaire from “foot” to “foot/ankle”. Subsequently, quantitative validation studies of the foot/ankle version were performed in patients with different foot and ankle problems [16–18]. The MOXFQ-Index was later suggested as a summary score based on the 16 items and intended to measure overall impact of foot and ankle problems on quality of life [18]. The MOXFQ has been translated and culturally adapted to multiple languages [19–28] and the measurement properties have been thoroughly tested in a patient population scheduled for surgery due to chronic foot or ankle conditions [14], where the treatment goal usually is focused on reducing disability through alleviating causes of chronic pain. In a scenario where patients undergo urgent procedures due to traumatic injuries, patients’ expectations and attention are aimed at recovering to their pre-injury health status. However, there are few validation studies assessing the performance of the MOXFQ in a non-elective context [29, 30]. Only recently, the content validity of the MOXFQ foot/ankle has been assessed in the context of surgically treated ankle fracture patients [31]. The Pain and Walking/Standing domains were acceptable for use in post-operative evaluation of patients that were allowed weight-bearing as tolerated. The Social Interaction domain had insufficient content validity for use in an ankle fracture population due to irrelevant items and ambiguous language. A summary score of the 16 items (the MOXFQ-Index), which was developed using higher order principal component analysis at a later stage [32], also had poor content validity as the items were not comprehensive in measuring overall impact of ankle fractures on health-related quality of life. However, pain and pain-related limitations are important concepts in the follow-up of ankle fracture patients, and the instrument is widely used in clinical practice. Therefore, despite previous findings of insufficient content validity for one of the three domains, demonstrating the measurement properties of this instrument holds clinical value. Another validation study comparing the four most used PROMs in ankle fracture research found that MOXFQ had the best measurement properties where data from 12 weeks after injury were used [33]. The primary aim of this study is to assess the structural validity and reliability of the MOXFQ foot/ankle in the context of a one-year post-operative ankle fracture population. The secondary aim is to provide PASS estimates to aid in the clinical interpretation of the instrument.

## Methods

### Participants

This study contained two sub-studies: (1) a pragmatic study with one-year cross-sectional follow-up data from 636 patients collected as part of the clinical routines of the department; and (2) a test-retest study conducted at the end of the study period which included 390 patients where time since surgery was at least one year. Patients were included if they were at least 18 years of age and admitted to Stavanger University Hospital for primary surgical treatment of an acute ankle fracture (AO/OTA 44). The cross-sectional study and test-retest study included patients that were admitted in the period January 2017 to December 2020 and from February 2018 to December 2020, respectively. Patients were excluded if they had cognitive impairment; suffered from a peri-implantar fracture; did not complete final treatment at Stavanger University Hospital; missing national identity number; or were not fluent in the Norwegian language. Cognitive or language impairments were assessed by the health staff at the outpatient clinics. For the test-retest study, patients’ electronic records were also searched for documentation of cognitive impairments, e.g. dementia, severe psychiatric illnesses, substance abuse or terminal illnesses.

### The Manchester Oxford Foot Questionnaire (MOXFQ)

The MOXFQ is a 16-item questionnaire used in the evaluation of patients undergoing foot and ankle surgery [15, 18] and consists of three domains: (1) a Pain domain with five items; (2) a Walking/Standing domain with seven items; and (3) a Social Interaction domain with four items. The response options are given on a five-point Likert scale, where 0 indicates no problems and 4 indicates severe problems. A score for each domain is calculated as the sum score of the items in that domain and transformed to a scale from 0 to 100, where a higher score denotes greater severity of problems. MOXFQ-Index is calculated using the same approach, except the sum score is obtained from all 16 items [32].

### Anchors

Anchor-based methods use an external patient-reported reference point, i.e., an anchor, to evaluate how the PROM score relate to the patients’ current state [13]. The A1 anchor (Table 1) asked about patients’ perception of outcome after surgery [34]. Patients selecting the response options “Good”, “Very good” or “Excellent” were categorized as having an ankle-specific acceptable symptom state one year after the ankle surgery and used in the calculation of PASS estimates.

**Table 1:** Anchor questions used to establish external reference points. The A1 anchor was included in the cross-sectional study. The A2 and A3 anchors were included in the test-retest study

Anchor	Question	Response options
A1	How would you describe the result of the operation?	“Excellent”, “Very good”, “Good”, “Fair”, and “Poor”
A2	How is the operated ankle now, compared to the last time you completed the questionnaires?	“Much better”, “A little better”, “About the same”, “A little worse”, and “Much worse”.
A3	In general, how would you describe your health the past two weeks?	“Changed” and “Unchanged”

Anchors were also used to assess that the responders did not have a change in their health state between measurements, which is important for the methodological quality of a test-rest study [35, 36]. Respondents that selected both the response option “About the same” on the A2 anchor and “Unchanged” on the A3 anchor were defined as stable in the test-retest study.

### Data collection

The data for the cross-sectional study were gathered as part of clinical follow-up routines. The MOXFQ and A1 anchor were sent to eligible patients by mail one year after surgery and returned in a prepaid envelope. Non-responders received a reminder after four weeks.

The data collection for the test-retest study was conducted in the period June 2022 to June 2023 with a time interval of at least two weeks between the measurements. Eligible patients received the MOXFQ by mail, and only responders of the first measurement (T1) were invited to respond to the second measurement (T2). The MOXFQ, A2 and A3 anchors were included in T2. The anchor questions were used to ensure stable patients, i.e., that the responders had not changed on the construct of interest between T1 and T2. Non-responders received one reminder telephonically and one reminder by paper for T1 and T2. The data were transferred to the database of the local fracture registry (Frakturregisteret i Helse Stavanger) [37–40] using automated forms processing [41].

### Statistical analysis

The assessment of the measurement properties followed the COSMIN study design checklist [36, 42–44]. In accordance with the MOXFQ user manual, missing data were not imputed since the study re-evaluates measurement properties [45]. The cross-sectional study only utilized data that contained complete item responses due to the assessment of structural validity. In cases of missing items in the test-retest study, the respondent’s remaining data on the relevant domain were omitted from the analyses.

#### Structural validity

The structural validity of the MOXFQ was assessed using confirmatory factor analysis (CFA). The model fit was evaluated using Tucker-Lewis Index (TLI), Comparative Fit Index (CFI), Root Mean Square Error of Approximation (RMSEA) and Standardized Root Mean Square Residual (SRMR), and obtained using weighted least squares means and variance adjusted estimation method to allow for the ordinal nature of the MOXFQ items [46]. The guidance values for a good model fit were values above 0.90 for TLI and CFI, and values below 0.060 and 0.080 for RMSEA and SRMR, respectively [47]. Model fit of alternative models were compared using Chi-squared tests.

Convergent validity reflects the degree to which the items within a domain measure the latent construct. Values above 0.50 for standardized estimates for factor loadings and for the average variance extracted (AVE) were considered acceptable [48].

Discriminant validity indicates the extent to which items intended to measure one construct, were not measuring another construct. A maximum shared variance (MSV) smaller than the AVE indicate acceptable discriminant validity [48]. Interdimensional correlation was calculated for the MOXFQ domain scores using Spearman’s Rho correlation. Values below 0.50 were considered weak correlations, 0.50 to 0.75 were moderate, and above 0.75 were considered strong.

The structure of the instrument was further explored with a unidimensional model and a second-order factor model to assess if the three domain scores could be reported as a summary score when used in the context of ankle fracture patients. Correlations above 0.80 between the first-order factors and the second-order factor indicate a high degree of shared variance between the factors and the reporting of a summary score may be justified [49]. An orthogonal bi-factor model was also applied to assess if the specific factors (MOXFQ-domains) provided unique variance when the variance from the general factor (MOXFQ-Index) was partitioned out [50]. Mean uniform factor loadings above 0.32 for a domain indicate that this domain would provide unique variance not reported by the summary score [51].

#### Reliability

Internal consistency, measurement error estimates and test-retest reliability were calculated for the assessment of reliability. Internal consistency was assessed by calculating the McDonald’s omega for each domain of the MOXFQ and the MOXFQ-Index using the data from the cross-sectional study. Values in the area 0.70–0.95 were considered acceptable [52].

The measurement errors of the MOXFQ domains and MOXFQ-Index were assessed by calculating the limits of agreement (LoA) and the standard error of measurement (SEM). A paired t-test of the mean scores of T1 and T2 was performed to assess if there were statistically significant (*p* < 0.05) systematic differences. The LoA was defined as the mean difference between T1 and T2 ± 1.96 x the standard deviation of the differences between T1 and T2 (SD_diff_) and plotted on a Bland and Altman plot. The SEM equals the square root of the total error variance [35].

Intraclass correlation coefficients (ICC) for the test-retest reliability were calculated for the MOXFQ domains and MOXFQ-Index using a single measurement two-way mixed-effects model with absolute agreement [53]. Values of 0.70 and above indicated sufficient reliability [52, 54].

#### Interpretability

The distribution of response frequencies was examined for ceiling or floor effect and defined as a clustering of best or worse possible scores of more than 15% in a domain, respectively [55]. PASS was estimated with the 75th percentile method using the scores for patients who replied “Good” or better on the A1 anchor [13]. The correlations of the A1 anchor with the domains and MOXFQ-Index were assessed with Spearman’s Rho. The 95% CIs were estimated using bootstrap (B = 1000) and presented as bias-corrected percentile intervals.

The descriptive statistics and reliability estimates were analyzed using IBM SPSS v. 29.0 and Stata v. 17. CFA was performed using R v. 4.4.0 with lavaan v. 0.6–19 [56]. The PASS estimates were calculated using Stata v. 17.

## Results

### Sample characteristics

**Fig. 1:**
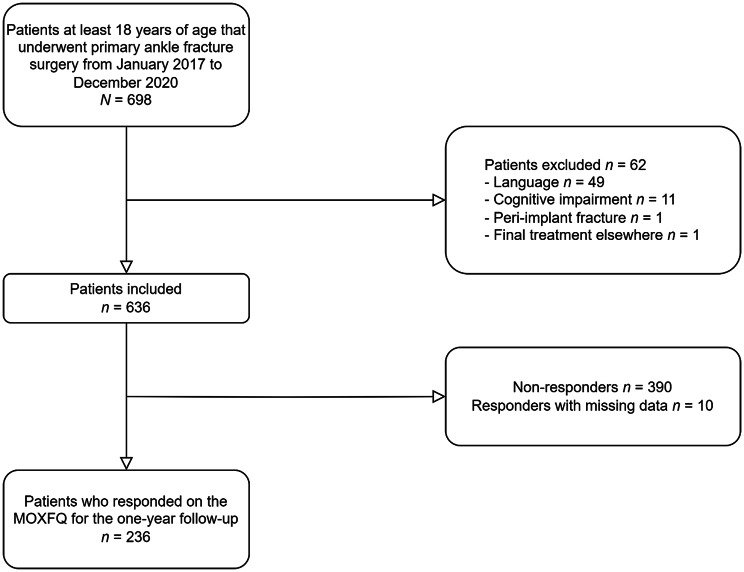
Flow chart illustrating patient inclusion and exclusion of the cross-sectional study

**Fig. 2:**
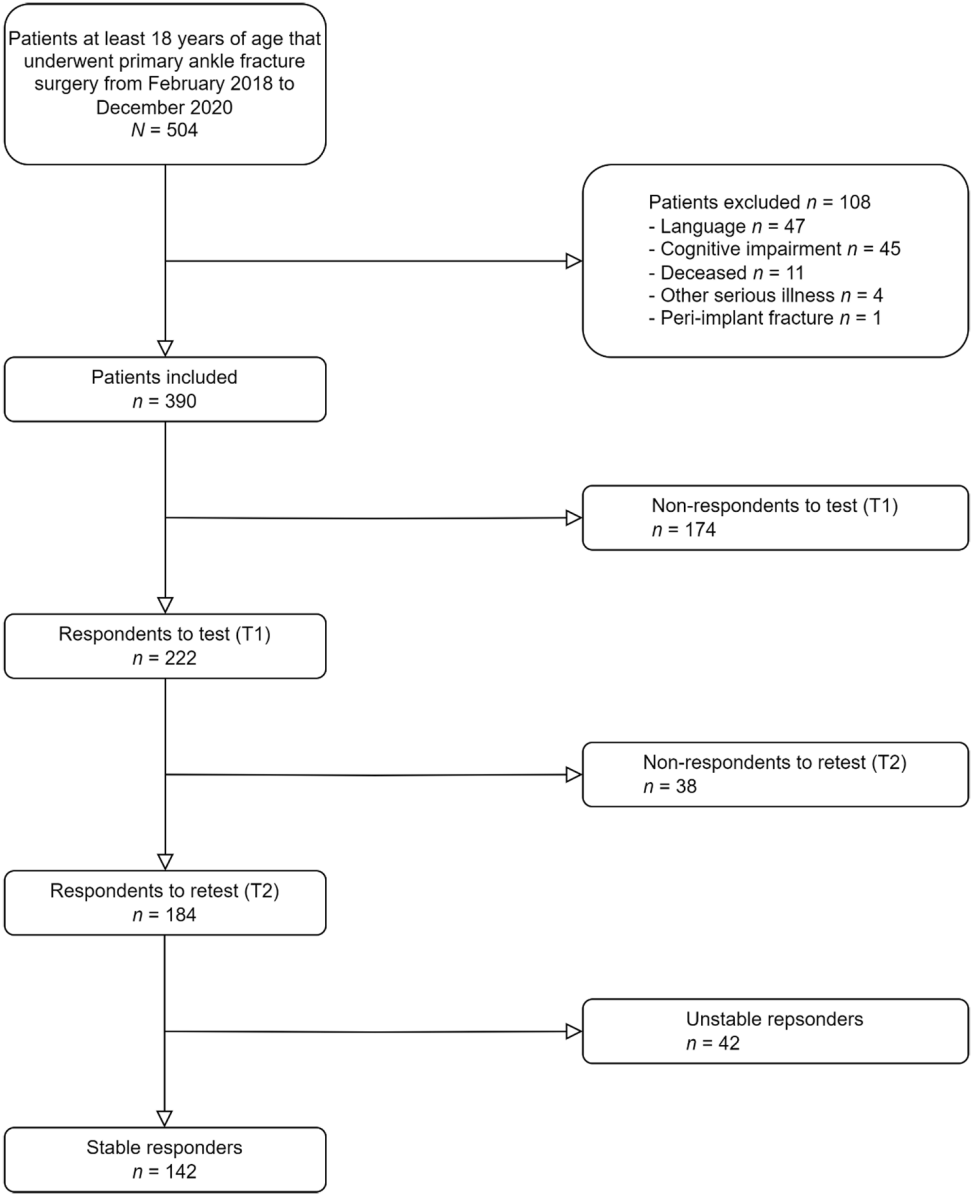
Flow chart illustrating patient inclusion and exclusion of the test-retest study

The study population for the cross-sectional study included 636 patients (Fig. 1). Ten respondents returned questionnaires with missing values. Complete MOXFQ was returned by 236 respondents (response rate 37.1%). The response rate for the test-retest study was 47.2% (184/390) (Fig. 2). One hundred and forty-two respondents (77.2%) reported that they were stable (A2 and A3) between the first (T1) and second time point (T2) of filling out the questionnaires. The mean time interval between T1 and T2 was 64 days (range 14 days to 182 days). Responders were older and more often women than non-responders (Table 2 and Additional file 1).

**Table 2:** Sample characteristics of patients in the cross-sectional study comparing the included patients (*n* = 636) and responders (*n* = 236)

Characteristics	Included patients *n* = 636	Responders *n* = 236	*P* value
Age years, mean (SD)	52.8 (17.2)	55.0 (15.2)	0.009
Sex, female	366 (57.5)	160 (67.8)	< 0.001
Side, right	332 (52.2)	122 (51.7)	0.844
ASA			0.159
1	220 (34.6)	72 (30.5)	
2	318 (50.0)	131 (55.5)	
3	75 (11.8)	28 (11.9)	
4	2 (0.3)	0 (0)	
Missing data	21 (3.3)	5 (2.1)	
AO			0.118
A	15 (2.4)	9 (3.8)	
B	449 (70.6)	169 (71.6)	
C	172 (27.0)	58 (24.6)	
Open fracture	16 (2.5)	6 (2.5)	0.974
Final treatment			0.263
ORIF	547 (86.0)	208 (88.1)	
Nail	23 (3.6)	5 (2.1)	
Screws, syndesmosis, tight rope	66 (10.4)	23 (9.7)	
External fixation as primary temporary treatment	41 (6.6)	19 (8.1)	0.259

Respondents completed the questionnaire in average 398 days (SD 36.8) after surgery. Data were given as n (%) unless otherwise stated. Responders and non-responders were compared using Pearson Chi-square tests except for age where an independent samples t-test was used

### Descriptive statistics

The item mean scores for the cross-sectional study were skewed towards the best possible scores (Table 3 and Additional file 2). The highest item mean score of 1.64 (SD 1.13) was found for item 15 (usual pain). The lowest scores were found for item 9 (self-conscious about foot/ankle); mean score 0.34 (SD 0.71) and item 10 (self-conscious about shoes); mean score 0.27 (SD 0.65). These two items were also high on skewness (2.4, 2.8) and kurtosis (6.5, 8.3), and had the most ceiling effect with 77% and 82% of responses for the best possible health, respectively.

**Table 3:** Descriptive statistics of item responses on the MOXFQ (*n* = 236) with mean, standard deviations (SD) and distribution of responses in the cross-sectional study

Domain/item^a^	Mean (SD)	Response category				
		0	1	2	3	4
Pain	31.8 (22.5)^b^					
Q1 Pain	1.61 (1.04)	17	26	37	17	3
Q11 Evening pain	1.38 (1.16)	31	21	32	12	4
Q12 Shooting pain	0.89 (0.95)	45	26	25	3	1
Q15 Usual pain	1.64 (1.13)	19	27	28	23	3
Q16 Night pain	0.85 (1.05)	53	18	22	5	2
Walking/Standing	28.9 (26.1)^b^					
Q2 Long distances	1.28 (1.22)	36	22	25	11	6
Q3 Way of walking	1.23 (1.08)	33	25	31	9	3
Q4 Walk slowly	1.15 (1.15)	39	25	22	11	3
Q5 Stop and rest	0.92 (0.99)	45	24	25	5	1
Q6 Hard/rough surfaces	1.09 (1.11)	39	27	21	9	3
Q7 Standing	1.13 (1.15)	41	21	22	13	2
Q8 Bus/car	0.94 (1.23)	54	17	15	8	6
Social Interaction	13.9 (16.7)^b^					
Q9 Self-conscious foot/ankle	0.34 (0.71)	77	15	6	1	1
Q10 Self-conscious shoes	0.27 (0.65)	82	11	5	1	0
Q13 Work/everyday	0.62 (0.92)	62	19	14	5	0
Q14 Social/recreational	0.98 (1.10)	46	23	22	7	3
MOXFQ-Index	25.5 (20.5)^b^					

The distribution of responses was given as percentage of responders for each category

^a^The wording of the items are in abbreviated forms

^b^Scoring presented on a scale 0 to 100 where 0 is best possible health

### CFA and model fit

**Fig. 3:**
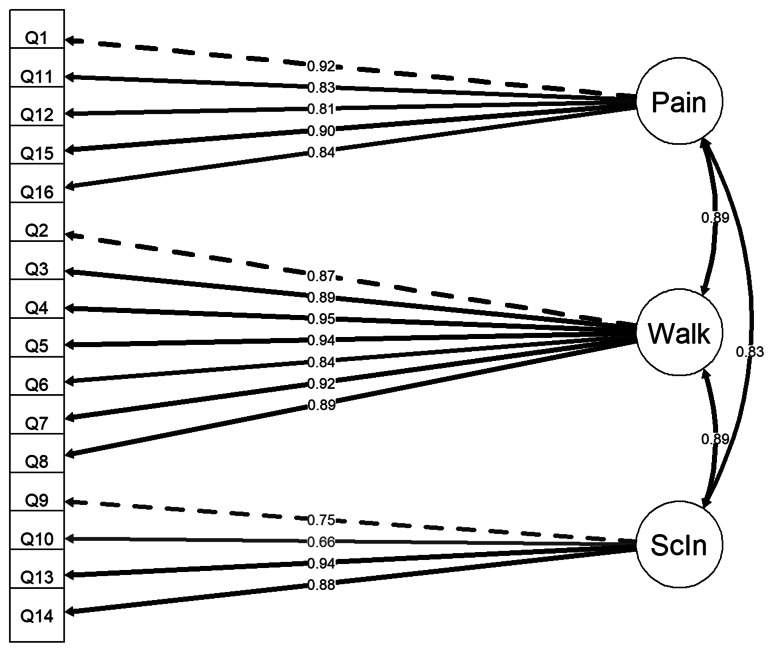
Three-factor model of the MOXFQ

CFA of the three-factor model of the MOXFQ (Fig. 3) demonstrated good model fit when assessed according to the guidance values (Table 4).

**Table 4:** Model fit values for the measurement models of the MOXFQ

Indices	Guidance values	Three-factor^a^	Second-order factor^a^	Uni-dimensional	Bi-factor
Tucker-Lewis Index (TLI)	> 0.90	0.94	0.94	0.88	0.95
Comparative Fit Index (CFI)	> 0.90	0.95	0.95	0.89	0.96
Root Mean Square of Approximation (RMSEA)	< 0.060	0.094	0.094	0.139	0.088
Standardized Root Mean Square Residual (SRMR)	< 0.080	0.039	0.039	0.055	0.032

^a^The second-order factor model is mathematically equivalent to the three-factor model

### Convergent and discriminant validity

The three-factor model demonstrated acceptable convergent validity with item factor loadings above 0.50 (Fig. 3). Items in the Walking/Standing and Pain domains had acceptable AVE values of 0.73 and 0.64, respectively. However, the Social Interaction domain had an AVE of 0.49, which was just below the threshold for acceptable convergent validity.

The discriminant validity of the MOXFQ domains were poor with correlations between the three factors reported to be between 0.79 and 0.88, indicating that they have a high degree of shared common variance (Fig. 3; Table 5). The MSV for all domains were reported to be higher than their respective AVE (Table 5). The interdimensional correlation between Pain and Walking/Standing was strong (0.84), and the Social Interaction domain correlated moderately with the Pain and the Walking/Standing domains with correlation coefficients of 0.69 and 0.77, respectively.

**Table 5:** Validity of the three MOXFQ domains

	AVE	MSV	Inter-factorial correlation		
			Pain	Walking/ Standing	Social Interaction
Pain	0.64	0.77^a^		0.88	0.79
Walking/ Standing	0.73	0.77^a^	0.88		0.88
Social Interaction	0.49^b^	0.77^a^	0.79	0.88	

*AVE* Average variance extracted, *MSV* Maximum variance extracted

^a^Discriminant validity issue (MSV larger than AVE)

^b^Convergent validity issue (AVE smaller than 0.50)

### Other measurement models

**Fig. 4:**
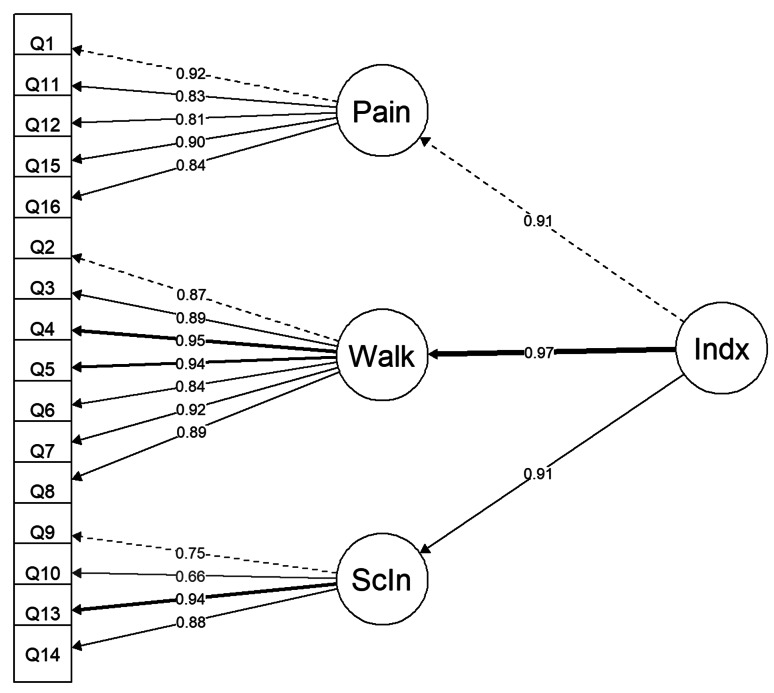
Second-order factor model of the MOXFQ

**Fig. 5:**
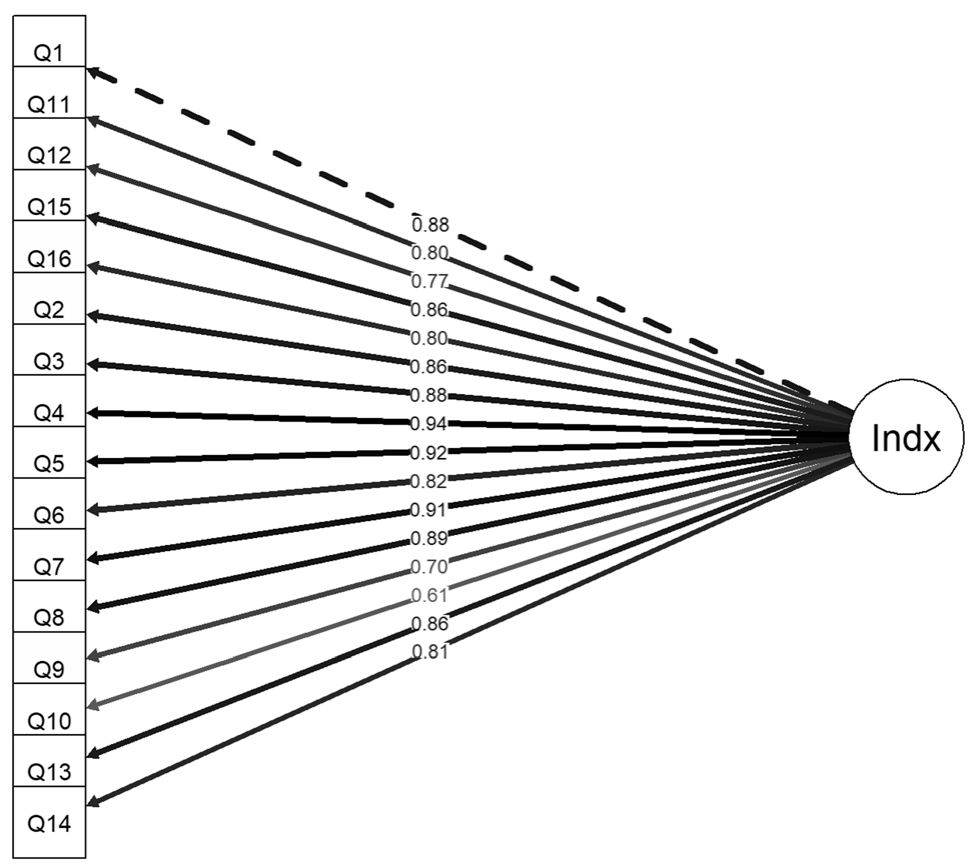
Unidimensional model of the MOXFQ with only one factor (MOXFQ-Index)

**Fig. 6:**
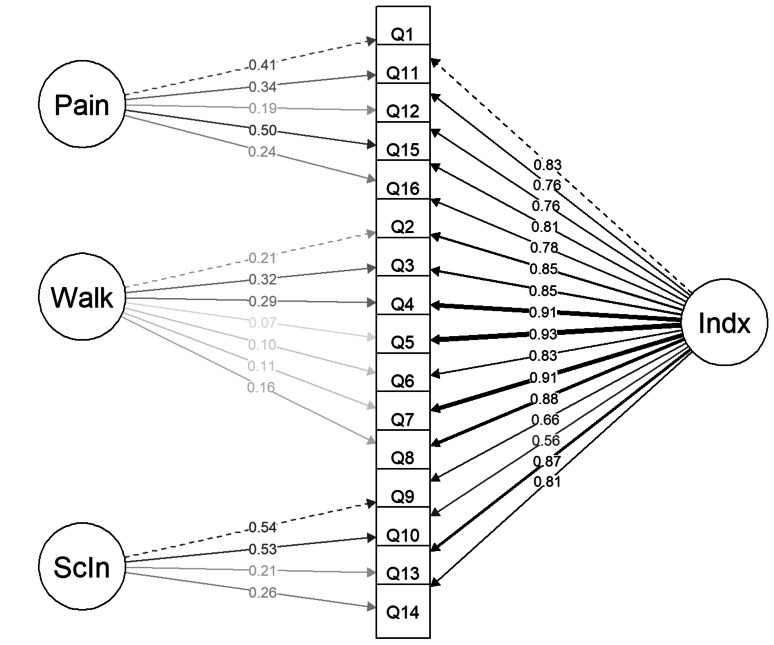
Bi-factor model of the MOXFQ with a general factor (MOXFQ-Index) and three specific factors (Pain, Walking/Standing and Social Interaction)

Correspondingly, the second-order factor model (Fig. 4) which is mathematically equivalent to the three-factor model and therefore indistinguishable in terms of model fit parameters and chi-squared values, showed substantial loadings off all three first-order factors (0.91–0.97) on the second-order factor (Table 6). The unidimensional model (Fig. 5) displayed worse model fit values than the other measurement models (Table 4), although with a high average factor loading of 0.83 (Table 6). The bi-factor model (Fig. 6) had the best model fit (Table 4). The average factor loading for the MOXFQ-Index in the bi-factor model (0.81) was close to the unidimensional model. However, the Pain domain and the Social Interaction domains also presented with substantial mean loadings of 0.33 and 0.38, respectively (Table 6). All model comparisons provided Chi-squared p-values that were highly statistically significant (*p* < 0.001).

**Table 6:** Mean factor loadings (range) for the measurement models of the MOXFQ

	Three-factor	Second-order factor	Uni-dimensional	Bi-factor
Pain	0.86 (0.81, 0.92)	0.86 (0.81, 0.92)	N/A	0.33 (0.19, 0.50)
Walking/Standing	0.90 (0.84, 0.95)	0.90 (0.84, 0.95)	N/A	0.18 (0.07, 0.29)
Social Interaction	0.81 (0.66, 0.94)	0.81 (0.66, 0.94)	N/A	0.38 (0.21, 0.54)
MOXFQ-Index	N/A	0.93 (0.91, 0.97)	0.83 (0.61, 0.94)	0.81 (0.56, 0.93)

### Internal consistency

McDonald’s omega values for the items in the three domains showed good internal consistency (Pain 0.90, Walking/Standing 0.95, Social Interaction 0.80). The MOXFQ-Index had a McDonalds’ omega of 0.96.

### Measurement error and test-retest reliability

The estimates for reliability are reported in Table 7. The LoA for the MOXFQ domains and MOXFQ-Index were plotted on Bland and Altman plots (Additional file 3). The paired t-test did not show statistically significant systematic differences in mean scores between T1 and T2 for the MOXFQ. The SEM values ranged from 5.5 to 7.5, and the test-retest reliability of the MOXFQ domains and MOXFQ-Index were satisfactory with ICC values of 0.80 to 0.92.

**Table 7:** The mean scores for the MOXFQ and the differences between the first and second measurement of the test-retest study with estimates of the standard error of measurement (SEM) intraclass correlation coefficient (ICC)

	*n*	T1 mean (SD)	T2 mean (SD)	T2-T1 (SD)	*P* value	SEM	ICC (95% CI)	
Pain	136	23.2 (22.7)	24.1 (23.0)	0.85 (9.1)	0.28	6.5	0.92 (0.89–0.94)	
Walking/Standing	137	17.9 (23.6)	18.2 (22.6)	0.31 (10.7)	0.73	7.5	0.89 (0.85–0.92)	
Social Interaction	137	8.5 (14.5)	8.4 (15.2)	-0.14 (9.5)	0.87	6.7	0.80 (0.73–0.85)	
MOXFQ-Index	135	17.4 (19.6)	17.7 (19.5)	0.36 (7.8)	0.59	5.5	0.92 (0.89–0.94)	

*SEM* Standard error of measurement, *ICC* Intraclass correlation coefficient

### Patient acceptable symptom state (PASS)

Table 8 shows the PASS estimates and the correlations of A1 anchor with the domains and the MOXFQ-Index. All correlations were moderate to strong, indicating that the A1 anchor was an acceptable external reference point for the calculation of the PASS estimates. Two hundred and twenty-four patients responded on the A1 anchor. 91% of the patients reported that the result of their surgery was good or better (Good 22%; Very good 46%; Excellent 22%).

**Table 8:** Spearman’s rho correlations between the A1 anchor and MOXFQ (*n* = 236), and the patient acceptable symptom state (PASS) estimated using the 75th percentile method (*n* = 203)

	Pain	Walking/Standing	Social Interaction	MOXFQ-Index
Correlation with anchor	0.60	0.60	0.47	0.63
PASS (95% CI)	45 (45, 50)	39 (36, 46)	19 (19, 25)	34 (33, 42)
*PASS* Patient acceptable symptom state				

## Discussion

### Main findings

This is the first study to assess the structural validity and reliability of the MOXFQ in an ankle fracture context one year after surgical treatment. The original three-factor measurement model demonstrated good structural validity. However, there was clearly an issue with the discriminant validity of the three highly correlated domains, suggesting that these domains measured overlapping constructs. Correspondingly, a mathematically equivalent second-order model demonstrated strong factor loadings of all three first-order factors onto a second-order factor. Further, a bi-factor analysis demonstrated a strong general factor but also unique variance in the Pain and Social Interaction domains. The assessment of the reliability confirmed good internal consistency for the domains and test-retest reliability of the domains and MOXFQ-index score. New PASS estimates were presented for this patient group.

### The validity of the original MOXFQ in an ankle fracture context

The structural validity should be assessed after the assessment of the content validity according to the COSMIN-approach [57]. The assessment of the structural validity in an instrument examines the unidimensionality of the domains and if the domains measure the constructs in a meaningful way, i.e., if the data appropriately fit the model. The MOXFQ was initially developed for patients that were scheduled for hallux valgus surgery, where the exploratory factor analysis defined three separate factors, i.e., three sets of items that measure three constructs [15]. The current study applied CFA to the original three-factor model for data obtained from patients one year after ankle fracture surgery and the analysis returned good model fit values. The three-factor structure also displayed good convergent validity, where the Pain domain and the Walking/Standing domain were able to explain the majority of the variance of their construct, and the Social Interaction domain was just at the threshold (AVE 0.49). These analyses indicate that the items within each domain were converging towards the same latent concept. However, the strong correlations between the factors in this model indicated poor discriminant validity, which was also supported by the high values of shared variance, i.e. the information retrieved from the domains overlapped with each other. Discriminant validity is important to ensure that the items measure a unique construct. Poor discriminant validity would indicate that items within a domain correlate more with constructs outside their intended domain. In the current study, all three factors had poor discriminant validity, which suggest an abundance of items measuring the same latent trait without providing additional information. This raises important practical issues with the instrument. From a responder’s point of view, this might reduce the feasibility of the instrument due to survey fatigue. From a clinical point of view, clinicians would like to know if the subscales provide unique information or if a summary score would be sufficient. Additionally, the factor loadings for items 9 (self-consciousness about the foot/ankle) and 10 (self-consciousness about footwear) were noticeably lower than the remaining items in the Social Interaction domain, although above the threshold value. These items were also shown to have poor relevance in a validation study of the MOXFQ’s content validity in ankle fracture patients [31]. Considering that these items also presented with substantial ceiling effects, they are potential candidates for removal in a modified version of the instrument.

### Exploring the factor structure of the MOXFQ

The use of the MOXFQ-Index score in an ankle fracture population demands further exploration of the factor structure. Presented with the poor discriminant validity of the three-factor model, a relevant query would be to examine the possibility of reporting the three domains as a summary score. The second-order factor model provided high loadings of the first-order factors (Pain, Walking/Standing and Social Interaction) onto the second-order factor (MOXFQ-Index), implying that the use of a summary score might be justified. Surprisingly, structuring a simple unidimensional model demonstrated substandard model fit values.

The bi-factor model had the best model fit values and provided insight into whether the reporting of the domain scores accommodated additional information when used together with the MOXFQ-Index. Figure 6 shows an average loading of 0.33 for the Pain domain and 0.38 for the Social Interaction domain, meaning that about 10–15% of the variance were unique to each of these domains. The Walking/Standing domain had an average factor loading of 0.18 i.e., very little unique variance, which suggests that the construct measured by the MOXFQ-Index would be similar to the construct measured by the Walking/Standing domain.

The exploration of the instrument’s structure with different models demonstrated that the 16 items are measuring a common underlaying trait, however, the data did not fit well to a unidimensional model, i.e., the solely use of summary score was not supported. Further exploration with a bi-factor model suggested that the domain scores should also be reported since the Pain- and Social Interaction domains contained additional information that was not captured by the MOXFQ-Index. However, the factor analyses also provided evidence that the Social Interaction domain may not be as relevant for the ankle fracture population as for hallux valgus patients. For example, the suboptimal convergent validity seen in the three-factor model suggests that the items were not entirely measuring the same construct with factor loadings of items 9 and 10 differing from items 13 and 14. Also, the bi-factor model revealed unique variance in this domain, although, the importance of this variance in ankle fracture patients is questionable given the stronger factor loadings of items 9 and 10 on the Social Interaction domain compared with the remaining items in this domain. Looking at the wording of these items, they are directed at worries related to footwear or appearance of the foot/ankle, while the remaining 14 items are pain related. These findings were also in concordance with a previous qualitative study assessing the content validity of the MOXFQ in ankle fracture patients [31]. Therefore, the Social Interaction domain score should seemingly not be used in the assessment of ankle fracture patients. In summary, reporting the scores of the Pain and Walking/Standing domains was justified. Much of the variance in the latter domain was captured by the MOXFQ-Index and reporting this score is in part redundant. However, considering its position in clinical use and clinicians’ desire for simple tools, its use might still be considered valuable.

### The applicability of reliability and measurement error estimates in clinical practice

PROMs are often used in clinical practice to measure the level of improvement or deterioration in a patient, and a high reliability of the measurement is important for the confidence in the observed scores. In general, a value of at least 0.70 has been considered an acceptable level of reliability on a group level [52, 58]. If one were to use the instrument in the evaluation of individual patients, higher reliability is necessary since any decision based on the instrument’s score will directly affect the patient, and values of at least 0.90 have been suggested as desirable [59]. Several of the ICC estimates reported in a systematic review of measurement properties of PROMs used in ankle fractures [29] superseded the threshold of 0.70. However, only one study of adequate methodology reported the 95% confidence interval (CI) [60]. The remaining studies had inadequate methodological quality [61, 62] or did not report the CI [63–65]. In the current study, an ICC of 0.92 (95% CI 0.89 to 0.94) was observed for the MOXFQ-Index. This indicates “good to excellent” reliability according to the guidelines by Koo et al. [53]. An important caveat is that this level of reliability only pertains to situations where the instrument is used in a similar context as the study was performed, i.e. in one-year postoperative ankle fracture patients.

The reliability of a measurement is closely connected with the level of measurement error. An important advantage of reporting the estimates of measurement error is that they are presented in units that are directly transferable to the instrument’s score, thereby providing the clinician with information on the level of uncertainty in the obtained score if used in clinical practice. The SEM derived from the current study permit clinicians to calculate the 95% CI of patients’ scores by taking the obtained scores and multiplying with ± 1.96*SEM.

The use of Bland and Altman plots are also helpful in the clinical interpretation of the instrument’s score. These plots (Additional file 3a–3d) demonstrate the systematic error between two measurements. The LoA provide information on how much the scores fluctuate between the first and second assessment, and about 95% of the variation will fall within these limits. Therefore, a change that is beyond these limits indicates a change beyond measurement error. The limits also represent the smallest change that can be detected by the instrument, i.e. the smallest detectable change (SDC). These limits should ideally be lower than the minimal important change (MIC), which is *the smallest change in score in the construct to be measured which patients perceive as important* [66], to be confident that a change score greater than the MIC is not due measurement error. These estimates would aid in the clinical interpretation of the scores and provide information on the ability of the instrument to detect a clinically meaningful change in the patient.

Other clinical cut-off values may also facilitate the interpretability of a PROM score, e.g. PASS. Thresholds for PASS in tibial plateau fractures [67] and upper extremity fractures [68, 69] has already been established. However, to the best of our knowledge, there is a lack of PASS estimates for ankle fracture patients, and the current study is the first study to report PASS estimates for the MOXFQ in an ankle fracture population. Such estimates are useful in the clinical evaluation of response therapy and may aid the clinician in the determination of successful treatment [70]. To completely validate the MOXFQ for use in the context of ankle fracture patients, the assessment of MIC, along with the assessment of the responsiveness of the instrument, should be an area of future research.

### Implications

The MOXFQ was developed to be used in pre- and postoperative evaluation of patients with a hallux valgus deformity, a chronic condition which more often affects females. On the other hand, patients suffering from an acute ankle fracture undergo urgent treatment, sometimes even emergent treatment, and have a bimodal distribution with a peak in young males and in elderly women [1]. Nonetheless, the instrument demonstrated good model fit values when used in the evaluation of ankle fracture patients. The results of this study imply that the domain scores should be reported with the MOXFQ-Index, as the domain scores contain some additional information not captured by the summary score. However, the current quantitative analyses disprove the use of the Social Interaction domain scores in the assessment of ankle fracture patients due to poor relevance, in concordance with a previous qualitative assessment. The study also provided reliability and measurement error estimates, in addition to PASS estimates to aid in the clinical interpretation of the scores.

Future research should focus on the optimization of the measurement model if the instrument should be used in ankle fracture patients, where especially items 9 and 10 proved to be problematic. As part of this, a detailed assessment of method variance [71] would be warranted. And importantly, the assessment of responsiveness and estimation of MIC are still lacking.

### Limitations

One of the major strengths with this study is the practical applicability of the results as the data collection was part of the clinical routines in patient follow-up. However, the pragmatic study design and the acute nature of the condition yielded a moderate response rate due to less rigorous routines for gathering data, e.g., the lack of accurate registrations of the patients’ cognitive- and language abilities. A systematic review reported response rates of 11.3–100% for PROMs used in orthopedic surgery with males, younger or older age as patient-specific factors that contribute to worse response rates [72], which was also reflected in the current study with statistically significantly more females and higher mean age in the respondent group (Table 2). Additionally, due to the pragmatic study design the A1 anchor was not worded as a precise domain-specific anchor. Although, the correlations between the anchor and the scores were acceptable (Table 8).

There were ceiling effects in many of the items, especially in the Social Interaction domain. From a clinical point of view, the treatment of ankle fractures aims to regain the physical function of the patients, and the ceiling effects found in the Pain and Walking/Standing domains were expected. The estimation method used handles skewed distributions by treating the indicators like ordinal variables.

A full investigation with the intent of improving the instrument would entail an account of method variance and item error correlations. We have not gone into this in the present paper, as the focus was how to use and interpret the instrument as presented.

## Conclusion

The MOXFQ demonstrated sufficient structural validity and reliability when used in the evaluation of a postoperative ankle fracture population. The exploration of the structure of the MOXFQ in our study revealed that the original three domains had items that measured overlapping constructs with strong inter-factor correlations and a strong general factor in the bifactor model. The latter model also revealed unique variance in the Pain and Social Interaction domains with little remaining variance in the Walking/Standing domain. However, the Social Interaction domain had suboptimal convergent validity, with stronger factor loadings of items 9 and 10 on the Social Interaction domain than the remaining items in this domain. In conclusion, reporting the scores of the MOXFQ-Index, Pain and Walking/Standing domains were justified from assessing the structure of the instrument. Interpreting the analysis together with previous qualitative findings, reporting the scores of the Pain and Walking/Standing domains alone are best supported when used in the follow-up of ankle fracture patients one year after surgery.

## Electronic supplementary material

Below is the link to the electronic supplementary material.

Supplementary Material 1

Supplementary Material 2

Supplementary Material 3

## Data Availability

The datasets used and/or analysed during the current study are available from the corresponding author on reasonable request.

## Abbreviations

AO/OTA: The Arbeitsgemeinschaft für Osteosynthesefragen / Orthopaedic Trauma Association
AVE: Average variance extracted
CFA: Confirmatory factor analysis
CFI: Comparative Fit Index
COSMIN: COnsensus-based Standards for the selection of health Measurement INstruments
ICC: Intraclass correlation coefficients
LoA: Limits of agreement
MIC: Minimal important change
MOXFQ: Manchester Oxford Foot Questionnaire
MSV: Maximum shared variance
PASS: Patient acceptable symptom state
RMSEA: Root mean square error of approximation
SDC: Smallest detectable change
SD_diff_: Standard deviation of differences
SEM: standard error of measurement
SRMR: Standardized root mean square residual
TLI: Tucker-Lewis Index
T1: First measurement in test-retest study
T2: Second measurement in test-retest study
