# Supplementary material for: Validity and reliability of the Manchester Oxford Foot Questionnaire (MOXFQ) in one-year postoperative ankle fracture patients—a validation study
Source: J Patient Rep Outcomes. 2025 Feb 5;9:14. doi: 10.1186/s41687-025-00845-w (PMC11799495; doi:10.1186/s41687-025-00845-w)
Supplement: Supplementary file 1 — Supplementary Material 1 [file 41687_2025_845_MOESM1_ESM.docx]

**Additional file 1** Sample characteristics of participants in the test-retest study comparing the included patients (*n* = 390), responders (*n* =184) and clinically stable responders (*n* = 142)

| **Characteristics** | **Included patients**  ***n* = 390** | **Responders**  ***n* = 184** | **Stable responders**  ***n* = 142** |
| --- | --- | --- | --- |
| Age years, mean (SD) | 52.0 (16.5) | 57.3 (14.8)* | 57.3 (14.6) |
| Sex, female | 229 (58.7) | 129 (70.1)* | 97 (68.3) |
| ASA |  |  |  |
| 1 | 150 (38.5) | 59 (32.1) | 72 (30.5) |
| 2 | 197 (50.5) | 102 (55.4) | 131 (55.5) |
| 3 | 35 (9.0) | 18 (9.8) | 28 (11.9) |
| Missing data | 8 (2.1) | 5 (2.7) | 5 (2.1) |
| AO |  |  |  |
| A | 11 (2.8) | 6 (3.3) | 5 (3.5) |
| B | 273 (70.0) | 133 (72.3) | 102 (71.8) |
| C | 106 (27.2) | 45 (24.5) | 35 (24.6) |
| Open fracture | 10 (2.6) | 6 (3.3) | 3 (2.1) |
| Final treatment |  |  |  |
| ORIF | 334 (85.6) | 161 (87.5) | 124 (87.3) |
| Nail | 9 (2.3) | 3 (1.6) | 2 (1.4) |
| Screws, syndesmosis, tight rope | 46 (11.8) | 20 (10.9) | 16 (11.3) |
| Respondents completed the questionnaires with a mean time interval of 64 days (range 14 to 182 days). Data were given as n (%) unless otherwise stated. Responders and non-responders were compared for statistically significant differences using Pearson Chi-square except for age where an independent sample t-test was used.  ^*^ = p < 0.05 | | | |
