# Supplementary material for: Validity and reliability of the Manchester Oxford Foot Questionnaire (MOXFQ) in one-year postoperative ankle fracture patients—a validation study
Source: J Patient Rep Outcomes. 2025 Feb 5;9:14. doi: 10.1186/s41687-025-00845-w (PMC11799495; doi:10.1186/s41687-025-00845-w)

**Additional file 2** Histograms showing the distribution of domain scores in the cross-sectional study for the MOXFQ and the MOXFQ-Index


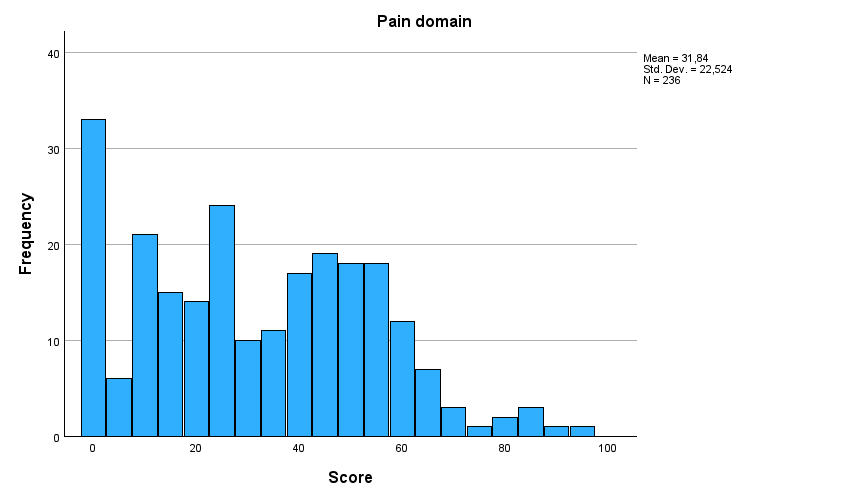


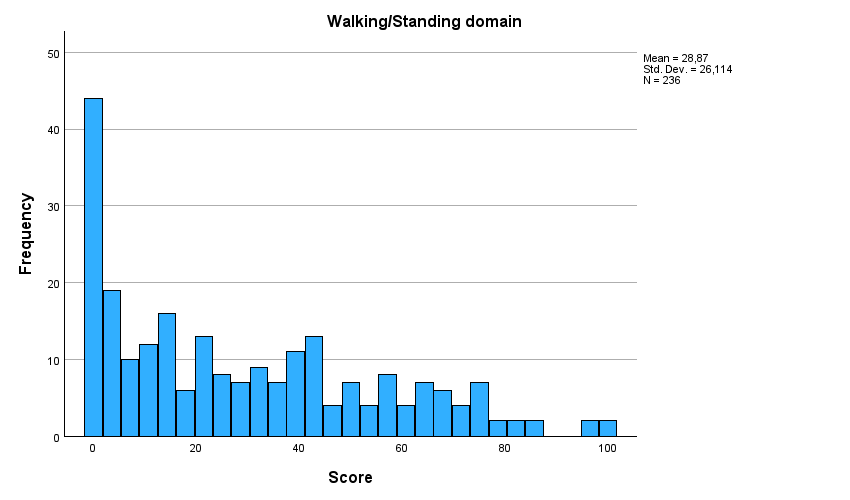


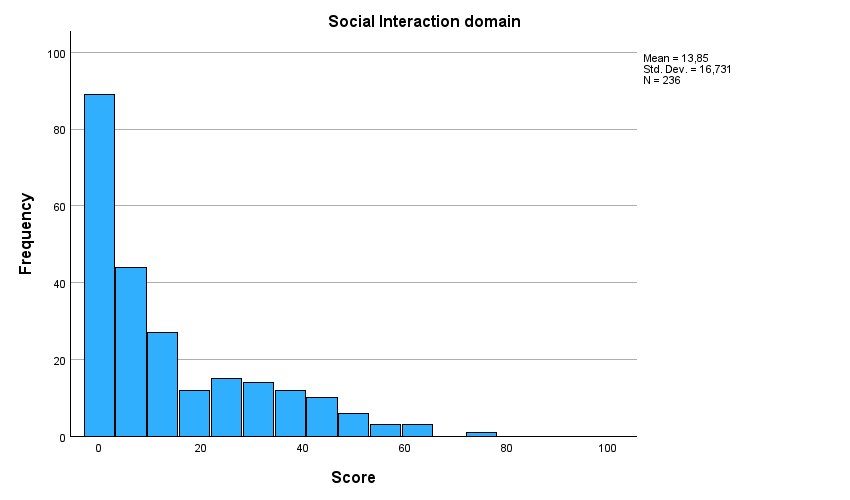


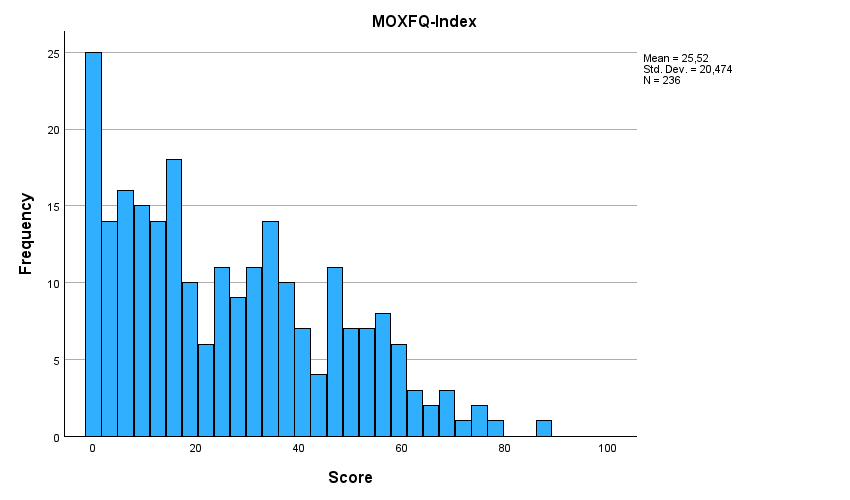

Supplement: Supplementary file 2 — Supplementary Material 2 [file 41687_2025_845_MOESM2_ESM.docx]
