# Supplementary material for: Validity and reliability of the Manchester Oxford Foot Questionnaire (MOXFQ) in one-year postoperative ankle fracture patients—a validation study
Source: J Patient Rep Outcomes. 2025 Feb 5;9:14. doi: 10.1186/s41687-025-00845-w (PMC11799495; doi:10.1186/s41687-025-00845-w)

**Additional file 3a** Bland and Altman plot with limits of agreement for the Pain domain


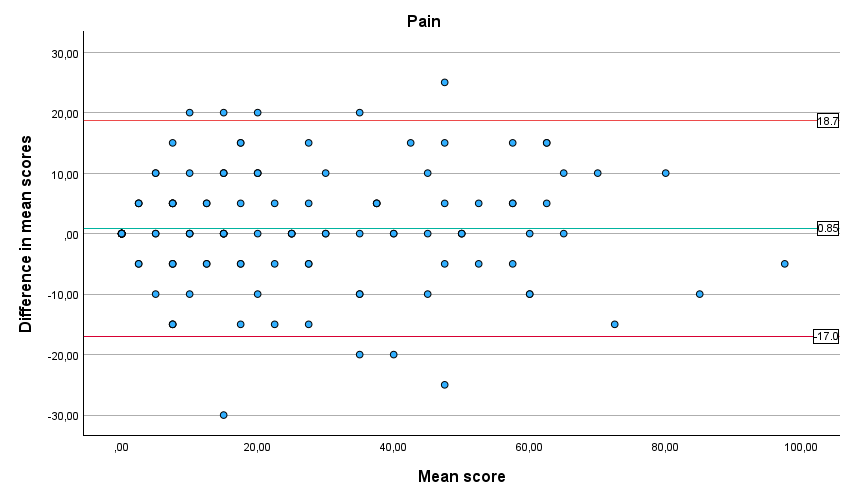


**Additional file 3b** Bland and Altman plot with limits of agreement for the Walking/Standing domain


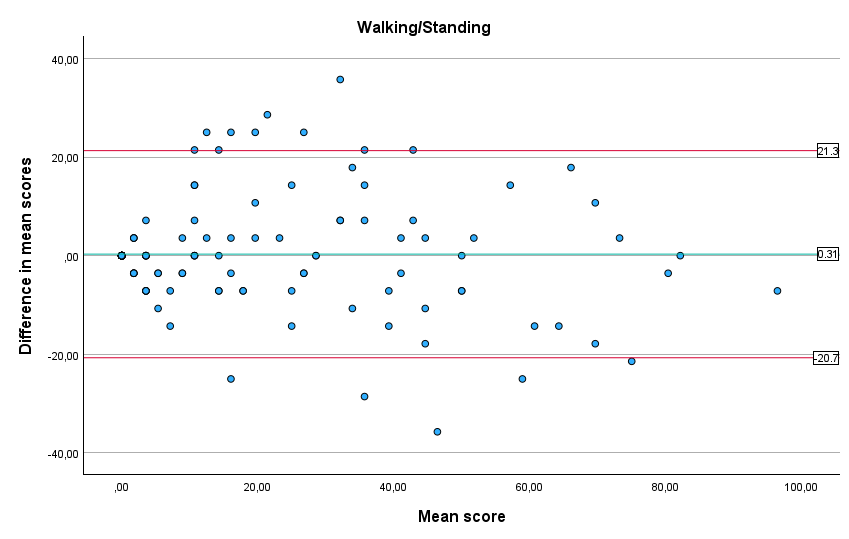


**Additional file 3c** Bland and Altman plot with limits of agreement for the Social Interaction domain


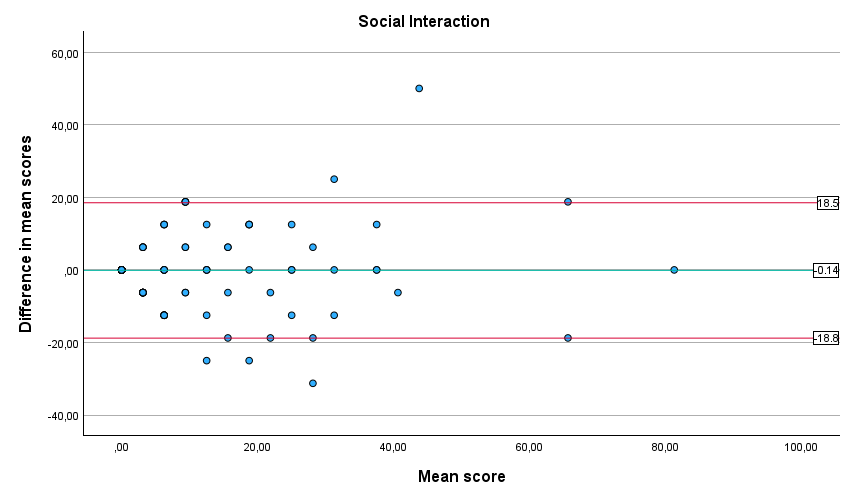


**Additional file 3d** Bland and Altman plot with limits of agreement for the MOXFQ-Index


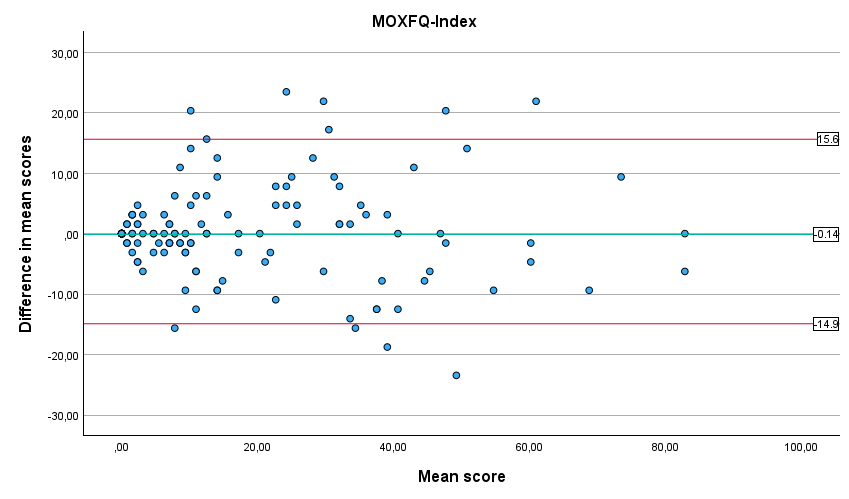

Supplement: Supplementary file 3 — Supplementary Material 3 [file 41687_2025_845_MOESM3_ESM.docx]
